# Simulation video: a tool to evaluate communications skills in radiologist residents

**DOI:** 10.1186/s12909-023-04582-w

**Published:** 2023-08-18

**Authors:** Ning Ding, Ge Hu, Xuan Wang, Hao Sun, Lan Song, Yu Chen, Daming Zhang, Huadan Xue, Zhengyu Jin

**Affiliations:** 1 Radiology Department, State Key Laboratory of Complex Severe and Rare Diseases, Peking Union Medical College Hospital, Chinese Academy of Medical Science and Peking Union Medical College, Shuai Fu Yuan 1#, Dongcheng Dist, Beijing, 100730 China; 2 Medical Research Center, State Key Laboratory of Complex Severe and Rare Diseases, Peking Union Medical College Hospital, Chinese Academy of Medical Science and Peking Union Medical College, Beijing, China

**Keywords:** Communication skills, Radiology education, Evaluation model, Simulation video

## Abstract

**Background:**

Effective communication is a crucial component of radiology resident training, and many different aspects need to be explored when teaching and evaluating communication skills. To ensure that radiology residents’ communication skill levels can be measured accurately, a standardized evaluation tool has been introduced. In twenty hospitals in Beijing, simulation videos have been developed as a way to assess the communication skills of radiology residents during their certification exams, to minimize evaluating biases. This study aims to assess the performance of a simulation video model in evaluating communications skills compared to the standard patient model.

**Methods:**

This is a retrospective observational study. The performance of standard patient and simulation video models was evaluated through an eight-year examination of communication skills in radiology residents. From 2014 to 2021, communications skill tests were administered to 1003 radiology residents in 20 hospitals in Beijing. The standardized patient (SP) model was applied in 2014, and simulation videos were used from 2015 to 2021. The difficulty and discrimination radio of the tests were evaluated. The subjective survey for candidates on two models of communication skills evaluation was performed and analyzed.

**Results:**

The simulation video model evaluation demonstrated stable difficulty (ranging from 0.92 to 0.98) and discrimination ratio (ranging from 0.37 to 0.49), except for minor exceptions of discrimination in 2019 (0.58) and 2020 (0.20). Furthermore, the Kruskal-Wallis H test revealed no significant differences in average scores between 2016 (93.9 ± 4.6) and 2018 (94.5 ± 4.2), 2016 and 2019 (97.3 ± 3.9), 2017 (97.0 ± 5.6) and 2019, 2017 and 2020 (97.7 ± 4.7), as well as 2019 and 2020 exams (all p ≥ 0.05). In addition, candidates who responded to the survey preferred the simulation video model (with a 77.2% response rate), with 62.7% choosing it over the SP model for communication skills evaluation.

**Conclusion:**

The simulation video demonstrated a stable and better acceptable construct for assessing radiology residents’ communication skills.

**Supplementary Information:**

The online version contains supplementary material available at 10.1186/s12909-023-04582-w.

## Introduction

Since the original version of Tomorrow’s Doctors in 1993 [1], medical education and resident training have shifted from a system based solely on time and process to one that emphasizes multiple competencies. This significant change has had a considerable impact on UK medical schools, many of which have started creating dynamic and innovative curricula inspired by the book’s guidelines.

Historically, radiologists have been primarily responsible for interpreting medical imaging and generating reports. However, with the shift towards multi-competency resident training in the medical field, evaluation tools became a new requirement for the profession.

Communication is one of the core competencies of radiology residents [2]. Effective communication is an essential aspect of providing high-quality patient care, and this applies to many medical subspecialties including radiology. While traditionally most medical imaging results are provided directly to the referring clinician, direct communication between radiologists and patients has become increasingly important, especially in situations such as direct interpretation of written reports to patients or interventional radiology [3]. The study of Gutzeit et al. showed that direct commutation from radiologists to patients after MRI examinations improved the radiology service and bonding between radiologists and patients [4].

Radiologists must communicate with colleagues, technicians, nurses, surgeons, internal physicians, and patients. The standardized patient (SP) model has a long history in medical training; it has played a role in professional medical teaching for more than 50 years [5, 6]. The first reported SP was coached by a neurologist to exhibit various neurological symptoms to assess the diagnostic skills of students. SP has also been applied to cultivate and evaluate communications skills for medical students [7–9] However, the training with SPs in evaluation can be time-consuming and hard to normalize, especially for large-scale evaluation, and it is difficult to obtain high reproducibility from different SPs [9].

How to assess communication skills has been challenging since the training on communication needs to be improved in both undergraduate and postgraduate education [10, 11]. Based on the Chinese national survey on radiology residency training, training programs mainly focus on patient care and medical knowledge rather than other competencies such as communication [10]. In China, resident training for radiology was nationalized in 2014, and all medical students looking to become radiology staff are required to complete a three-year residency training in radiology. This requirement is mandatory, irrespective of whether the medical student has achieved a bachelor’s, master’s, or doctoral degree, and was in place at the time of the study being conducted. To date, there is no national resident certification exam in China. Twenty hospitals in Beijing experience is the most advanced and representative in the country [10]. There are twenty hospitals in Beijing qualified as radiological residents standardized training centers, which are Peking University Third Hospital,Peking University First Hospital, Peking University People’s Hospital, Peking University Cancer Hospital, Beijing Jishuitan Hospital, Beijing Tsinghua Changgung Hospital, Beijing Hospital, Beijing Chaoyang Hospital Affiliated to Capital Medical University, Beijing Shijitan Hospital Affiliated to Capital Medical University, Beijing Tiantan Hospital Affiliated to Capital Medical University, Beijing Tongren Hospital Affiliated to Capital Medical University, Beijing Friendship Hospital Affiliated to Capital Medical University, Xuanwu Hospital of Capital Medical University, Chinese People’s Liberation Army Air Force Special Medical Center, Sixth Medical Center, General Hospital of Chinese People’s Liberation Army, The First Medical Center of the Chinese People’s Liberation Army General Hospital, Peking Union Medical College Hospital, Chinese Academy of Medical Sciences, Chinese Academy of Medical Sciences Cancer Hospital, Sino-Japanese Friendship Hospital and Beijing Aerospace General Hospital. Beijing’s overall radiology resident capacity has considered the significance and taken action to cultivate communications skills. Although there are studies about simulation training and evaluation in radiology resident communication skills [12, 13], no one has used simulation video in communication skill evaluation. Considering the shortcomings of the SP model for communications skills evaluation, the evaluation team initiated the simulation video model. The 2014 radiology resident certification exam initially used SP conversation for communications skill evaluation. In the SP test, each resident had a medical inquiry with a trained SP and received a score from two examiners. From 2015 to 2020, the novel simulation video was used. This retrospective study compared the advantages and disadvantages of the two evaluation models.

## Methods

This is a retrospective observational study. The objective and subjective evaluations were both performed. The performance of standard patient and simulation video models was evaluated through an eight-year examination of communication skills in radiology residents. From 2014 to 2021, communications skill tests were administered to 1003 radiology residents in 20 medical hospitals in Beijing. The standardized patient (SP) model was applied in 2014, and simulation videos were used from 2015 to 2021. The average score, difficulty, and discrimination radio of the tests were evaluated. The subjective survey for candidates on communication skills was performed and analyzed.

### The standardized patient conversation evaluation model

The evaluation process was designed in four phases. The first phase was to write the script, which included the medical history of the SP, the emotional status, and the primary concern. In the second stage, the SPs are recruited according to the following standards (Table 1). The SPs do not need to have a professional medical background. Standardized training was carried out for all SPs enrolled. The training content included fundamental medical knowledge and doctor–patient communication knowledge and skills. The training goal is to “be able to truly show the complaint”, which means to act out symptoms and chief complaints, such as headaches, and stomachaches. The third phase was the evaluation of the SP after the training to ensure that they met the requirements.The fourth stage was the final examination. The whole process was observed and independently evaluated by two examiners. Each resident had 10 min to communicate with the SP.

**Table 1:** SP Recruitment criteria

SP Recruitment criteria
1. Men and women ages 20–62
2. A certain level of education, a junior college degree(≥ three years) is required
3. Regular free time to cooperate with training and teaching
4. Good physical and mental health, good memory, and solid role-playing skills were prioritized

### Simulation video evaluation

The evaluation tool was designed in three phases, and the first phase was to write a script. The topics included breaking bad news, interpreting imaging reports, or taking a medical history. Bad communication practices were incorporated into the video in spoken words, nonverbal expression, tone of voice, body gestures, professional value, and attitude toward the patient. The second phase was to film a 5-minute video based on the script. The third phase was the final evaluation in which the short video was shown to the candidate, and the candidate was asked to indicate the bad practices that should be avoided. The final evaluation lasted for 10 min.

The standard for evaluation shown in Table 2 were applied in both SP and simulation video models. The items were scored, and the average score, difficulty, and distinction of the test were analyzed. Two interviewers rated one resident with a minimum working experience of ten years in radiology independently, and the final score was the average of the two scores. The total possible score was 120.

**Table 2:** Evaluation item and scale of marks

Examination item	Examination details	Scale of marks
Whether attention has been given to individual appearance	Appearance: Wheather dressed neatly with a professional demeanor (the white coat, tie, and shoes)	10: Clearly, describe what went wrong and what went right 8: Generally, describe what is wrong and what is right 6: Partially describe what is wrong and what is right 4: Slight reference to what is wrong and what is right 2: Little knowledge of what is wrong and what is right
Whether attention has been given to polite language	Words: Whether used polite language (which should include “thank you”, “you”, “please”, “hold on”, etc.)	
	Tones: Whether the tone of voice is neither humble nor overbearing, calm and clear	
Whether attention has been given to physical gestures	Whether shows empathy with physical gestures, such as forcefully closing the door, hugging arms while communicating with patients	
Patient Privacy	Whether give private room for patients changing clothes	
Whether attention has been given to optimizing the operation process	Whether gives patients appropriate recommendations and instruction before and during the examination	
Professional terms	Whether uses professional and understandable words	
Whether attention has been given to avoid nonmedically related behaviors	Whether picks up personal phone calls or chats with colleagues during the examination	
Whether attention has been given to radiation protection	Whether modulates best scan protocol and uses lead to shield non-illuminated fields	
Whether attention has been given to the feelings of patients, whether the reasonable requirements of patients were met	Whether the radiologist adequately answers patients’ questions regarding the examination and adjusts the scan parameters to promote better patient cooperation	
Strong sense of responsibility, respect for patients, and medical work	Whether the radiologist pursues an adequate diagnosis, regardless of the amount of effort and time required	

### Measures and statistical analyses

The data analysis and statistics were performed with Statistical Program for Social Sciences (SPSS) software, version 25.0.

The final performance score of any candidate was the average score of the two evaluators. The difficulty and discrimination ratios were calculated each year. The difficulty was calculated as D = M/F, D = difficulty, M = mean score of all candidates, and F = whole test scores.

The discrimination ratio was calculated as DR=(XH-XL)/N(H-L), DR = discrimination ratio, XH = total sum of the scores of the high-score group, high-mark group = top 27% candidates, XL = total sum of the scores of the low-score group, N = 27% of all candidates, H = highest score, L = lowest score.

The Kruskal-Wallis H test was used to perform pairwise comparison of the average scores between any two years using the simulation video model. In this study, statistical significance was determined at a P < 0.05.

## Results

### Objective evaluation: performance of the eight-year communication skills examination

A total of 1003 candidates completed the communications skills assessment from 2014 to 2021, and 99 candidates in 2014 underwent the SP assessment. From 2015 to 2021, 904 candidates underwent the simulation video assessment.

**Fig. 1:**
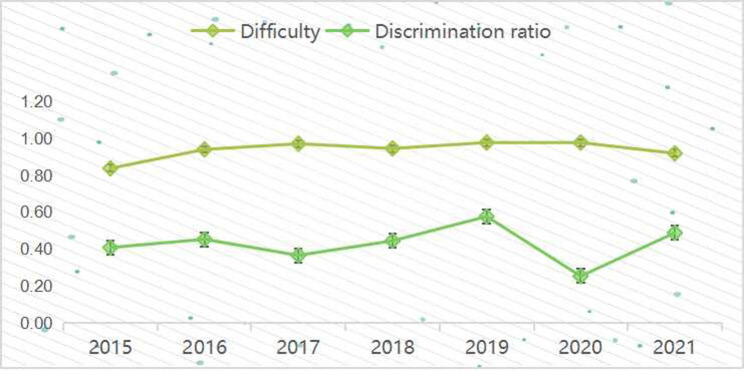
Annual performance discrimination ratio and difficulty from 2015 to 2021

Overall, 47.4% of the candidates had a bachelor’s degree, 37.2% had a master’s degree, and 18.4% had a doctorate degree. The age range for radiologists in Beijing to take the board examination was 24–32 years old. For SP model in 2014, the average score, difficulty and disicrimination ratio is 86.7 ± 13.4, 0.87 and 0.95.The difficultly and discrimination ratio of simulation videos was relatively stable from 2015 to 2021, with a slight exception in 2019 and 2020 (Fig. 1). Simulation vedio model annual performances of average score, difficulty and discrimination ratio are listed in Table 3. The pairwise comparison using Kruskal-Wallis H showed that there was no significant difference in the average scores between 2016 and 2018, 2016 and 2019, 2017 and 2019, 2017 and 2020, as well as 2019 and 2020 exams (all p ≥ 0.05)(Table 4).

**Table 3:** Annual performance of the communications skills evaluation (average of two evaluators)

Years	Number of candidates	Average score	Difficulty	Discrimination ratio
2014	99	86.7 ± 13.4	0.87	0.95
2015	122	83.7 ± 8.0	0.84	0.41
2016	138	93.9 ± 4.6	0.94	0.45
2017	152	97.0 ± 5.6	0.97	0.37
2018	132	94.5 ± 4.2	0.94	0.45
2019	73	97.3 ± 3.9	0.98	0.58
2020	160	97.7 ± 4.7	0.98	0.25
2021	127	91.8 ± 4.2	0.92	0.49

**Table 4:** Kruskal-Wallis H test of the annual score for pairwise comparison

P value	2015	2016	2017	2018	2019	2020
2015	——	——	——	——	——	——
2016	P < 0.001*	——	——	——	——	——
2017	P < 0.001*	P = 0.012*	——	——	——	——
2018	P < 0.001*	P > 0.999	P = 0.001*	——	——	——
2019	P < 0.001*	P = 0.079	P > 0.999	P = 0.016*	——	——
2020	P < 0.001*	P < 0.001*	P > 0.999	P < 0.001*	P > 0.999	——
2021	P < 0.001*	P < 0.001*	P < 0.001*	P = 0.001*	P < 0.001*	P < 0.001*

* means P<0.05.

### Comunication skills survey of the candidates

In total, 657 residents participated in the communication skills survey (77.2% response rate) in 2014–2021(Table 5). Most radiology residents were aware of the communications skills training (78.4%). However, only half had access to communications skills training, which means the communication teaching is incorporated into the candidate’s yearly learning program, or the candidate took the initiative to learn the knowledge and skills of communication. Most residents preferred the simulation video model (62.7%).

**Table 5:** Communication skills survey feedback

Survey questions	Agree	Disagree
I was aware of the communications skills training before the exam	78.4% (515/657)	21.6% (142/657)
I have received communications skills training before the exam	54.8% (360/657)	45.2% (297/657)
I was aware of how to receive communications skills training	51.9% (341/657)	48.1% (316/657)
Have you practiced with standardized patients or been assessed?	57.5%(378/657)	42.5%(279/657)
Do you think simulation video is better than the standardized patient model in communications skills evaluation?	62.7%(343/547)	37.3%(204/547)

## Discussion

As far as we know, the evaluation of communication skills among radiology residents over eight years in 20 hospitals in Beijing is the most comprehensive standardized evaluation reported to date. According to the quantitative analysis of the average score, difficulty, and discrimination ratio, using the simulation video model resulted in a stable evaluation of communication skills. The subjective assessment of candidates via a survey demonstrated that the simulation video model was more acceptable than the SP model.

While most of the average scores of the simulation video model from 2015 to 2021 were relatively stable, statistical analysis revealed that the scores in 2015 and 2021 differed significantly from those in other years. Several factors played a role in influencing the evaluation scores in 2015 and 2021. The adoption of the newly developed simulation video model test in 2015 required candidates to adjust to this new evaluation form, which could have affected their performance. In 2016, one year after introducing simulation vedio model into communication skill evaluation, the candidates demonstrated more excellent communications skills than they did in 2015 under similar test difficulty. Additionally, in 2021, the clinical rotations of residents who took the exams were disrupted for over six months due to the COVID-19 pandemic, which could have affected their preparation and performance.

The result of the survey showed that nearly half of the radiology residents do not have convenient access to appropriate communications skills training. The unpopularity of communications skills training may not be unique to radiology, but in many other medical specialties, novel trials advancing communications skills training for Chinese doctors are emerging [14–16]. Nevertheless, these attempts with limited sample sizes show a long way to go to popularize communications education.

Standardized patients have been used in medical education to improve communication skills [17–19] and in many other subspecialty training, such as pre-anesthetic assessment [15] or counseling of community pharmacists [8]. We initially used the standardized patients for evaluation in 2014; in that year, with nearly 100 candidates, four standardized patients were included in the final evaluation, and the recruitment and training process was time-consuming. Compared to the simulation video modality, the higher discrimination ratio of the SP model indicated heterogeneity among SPs. It was understandable that every SP has his or her unique communication style and subjective judgment. In that sense, the setting of different SPs in one evaluation may comprise its fairness and standardization. Compared with SP, simulation videos can quickly achieve the goal of fairness with higher stability. We collected feedback from the candidates, and the majority perceived that the simulation video model was better than the SP model in assessing communications skills. The performance in the first year (2015) of using the simulation video model was significantly lower than that of the following years (2016–2021, p < 0.001), even with the lowest difficulty level. This may be due to the maladjustment to the novel test method. However, with more experience for both the assessment team and the candidates, performance remained stable in the years following the introduction of the simulation video model (2016–2021).

Based on the annual performance of the evaluation, the application of the evaluation itself was a powerful intervention for improving awareness and mastery of communications skills among the radiology residents. From the residency candidates’ perspective, even with a little formal training, communications skills can be improved by self-reflection [20, 21] or observation and learning from senior colleagues [22, 23].

Our communications skills evaluation model was based on real-life hospital scenarios, The scoring was based on the knowledge that the doctor can have appropriate responses during patient-doctor communication only when he or she can pick up on clues from the patients. The original intention for implementing the communication evaluation was to use deliberately poor behavior as a mirror to inspire the candidates’ reflection on the proper patient-doctor relationship. We are convinced that residents will take this reflection back to their daily work environments as a toolkit for proper action and benefit daily from patient-doctor communication. Further study will be performed to evaluate the effect of videos demonstrating good doctor-patient interactions incorporated into the communication training program. Moreover, we also intended to carry out an individual improvement education program based on the trainees’ real-life communication scenarios.

To conclude, after eight years of experience, the simulation video model showed better acceptance and stability in assessing communication skills among radiology residents. It could be used as a benchmark for evaluating and training communication skills in other medical specialties.

### Electronic supplementary material

Below is the link to the electronic supplementary material.

Supplementary Material 1

## Data Availability

The datasets used and analyzed during the current study are available from the corresponding author upon reasonable request.
