# Supplementary material for: Simulation video: a tool to evaluate communications skills in radiologist residents
Source: BMC Med Educ. 2023 Aug 18;23:586. doi: 10.1186/s12909-023-04582-w (PMC10439603; doi:10.1186/s12909-023-04582-w)
Supplement: Supplementary file 1 — Supplementary Material 1 [file 12909_2023_4582_MOESM1_ESM.docx]

List of Beijing radiological resident standardized training centers

Peking University Third Hospital

Peking University First Hospital

Peking University People's Hospital

Peking University Cancer Hospital

Beijing Jishuitan Hospital

Beijing Tsinghua Changgung Hospital

Beijing Hospital

Beijing Chaoyang Hospital Affiliated to Capital Medical University

Beijing Shijitan Hospital Affiliated to Capital Medical University

Beijing Tiantan Hospital Affiliated to Capital Medical University

Beijing Tongren Hospital Affiliated to Capital Medical University

Beijing Friendship Hospital Affiliated to Capital Medical University

Xuanwu Hospital of Capital Medical University

Chinese People's Liberation Army Air Force Special Medical Center

Sixth Medical Center, General Hospital of Chinese People's Liberation Army

The First Medical Center of the Chinese People's Liberation Army General Hospital

Peking Union Medical College Hospital, Chinese Academy of Medical Sciences

Chinese Academy of Medical Sciences Cancer Hospital

Sino-Japanese Friendship Hospital

Beijing Aerospace General Hospital

北京大学第三医院

北京大学第一医院

北京大学人民医院

北京大学肿瘤医院

北京积水潭医院

北京清华长庚医院

北京医院

首都医科大学附属北京朝阳医院

首都医科大学附属北京世纪坛医院

首都医科大学附属北京天坛医院

首都医科大学附属北京同仁医院

首都医科大学附属北京友谊医院

首都医科大学宣武医院

中国人民解放军空军特色医学中心

中国人民解放军总医院第六医学中心

中国人民解放军总医院第一医学中心

中国医学科学院北京协和医院

中国医学科学院肿瘤医院

中日友好医院

北京航天总医院
